# Determination of a possible relationship between a single nucleotide polymorphism (SNP) in the promoter region of the *SIRT1* gene and production and reproduction traits in the Agerolese cattle breed

**DOI:** 10.5194/aab-62-107-2019

**Published:** 2019-03-22

**Authors:** Maria Selvaggi, Claudia Carbonara, Francesca Ciotola, Sara Albarella, Giulio Aiudi, Vincenzo Tufarelli, Cataldo Dario

**Affiliations:** 1 Department of Agricultural and Environmental Science, University of Bari Aldo Moro, Bari, Italy; 2 Department of Veterinary Medicine and Animal Production, University Federico II, Naples, Italy; 3 Department of Veterinary Medicine, University of Bari Aldo Moro, Bari, Italy; 4 Department of DETO, Section of Veterinary Science and Animal Production, University of Bari Aldo Moro, Bari, Italy

## Abstract

Sirtuins (sir2-like proteins) belong to the family of class III NAD${}^{+}$-dependent histone deacetylases. Among them, SIRT1 is the most studied sirtuin. It plays a key role in many biological processes in the liver, adipose tissue, muscle, pancreas, testis, ovary and mammary gland. It has an important function in endocrine signaling, with a specific role in glucose and fat metabolism. To date, in bovine species, only few SIRT1 single nucleotide polymorphisms (SNPs) have been reported in the literature. Thus, the aims of the present study were to estimate the allele and genotype frequencies at the g.-274C > G locus in the promoter region of the SIRT1 gene and to investigate, for the first time, the relationship among different genotypes and milk production and some reproduction traits in a sample of cows belonging to the Agerolese breed. All the animals were genotyped at the abovementioned locus using the PCR-RFLP technique. The investigated population was found to be polymorphic at the investigated locus. Concerning milk production performances, significant differences between genotypes were found in daily milk yield (DMY), fat percentage (FC), lactation length (LL), peak daily milk yield (PY) and 305-day milk yield (305MY). Moreover, the effect of the g.-274C > G genotype on age at first calving and calving period was significant. In conclusion, our findings are promising and should encourage scientists to further investigate the effect of genetic polymorphism of sirtuins on milk performance and reproductive traits.

## 1 Introduction

Sirtuins (sir2-like proteins) are mammalian homologs of the silent information regulator 2 (sir2) gene of *Saccharomyces cerevisiae* and they belong to the family of class III NAD${}^{+}$-dependent histone deacetylases (HDAC III) (Frye, 1999; North and Verdin, 2004).

The mammalian sirtuin family consists of seven members (SIRT1-7). Each sirtuin is characterized by a conserved 275 amino acid catalytic core domain and peculiar N-terminal and/or C-terminal sequences of variable length (Michan and Sinclair, 2007). Mammalian sirtuins also differ in their subcellular localization. SIRT1, SIRT6 and SIRT7 are predominately in the nucleus, but SIRT1 also shows important cytoplasmic functions. SIRT2 resides most prominently in the cytoplasm, while SIRT3-5 are mitochondrial sirtuins (Michan and Sinclair, 2007; Michishita et al., 2005; North et al., 2003; Vaziri et al., 2001).

Sirtuins shows a gene silencing activity by deacetylating nucleosomes to form heterochromatin; moreover, they act as sensors of the cellular energy status, establishing a link between gene silencing and metabolism in vivo (Braunstein et al., 1993; Denu, 2003).

SIRT1, the most investigated sirtuin, shows a key role in many biological processes in the liver, adipose tissue, muscle, pancreas, testis, ovary and mammary gland to regulate gene transcription, DNA repair, genome stability, cell proliferation, cell survival and apoptosis, and energy metabolism. SIRT1 interacts with several transcription factors in accordance with its role in gene silencing and heterochromatin formation (Vaquero et al., 2004; Shoba et al., 2009; Revollo and Li, 2013). It has an important function in endocrine signaling, specifically in the glucose and fat metabolism inducing the transcription of several genes involved in metabolism (Li et al., 2013a). In fact, SIRT1 promotes the expression of gluconeogenic genes in the liver, fatty acid oxidation in muscle and fat mobilization in adipose tissue (Picard et al., 2004; Gerhart-Hines et al., 2007; Rodgers and Puigserver, 2007). Moreover, SIRT1 is expressed in the mammary gland where it modulates the efficacy of estrogen-IGF1 signaling and regulates the timing of ductal morphogenesis during mammary gland development in mice (Li et al., 2007).

In the bovine species, the SIRT1 gene has been assigned to chromosome 28; its coding region consists of nine exons (Ghinis-Hozumi et al., 2011). To date, in bovine species, only few SIRT1 polymorphisms have been reported in the literature. In particular, Li et al. (2013a, b) revealed the presence of five single nucleotide polymorphisms (SNPs), namely g.-382G > A, g.17324T > C, g.17379A > G, g.17491G > A and g.-274C > G. All these SNPs were located in noncoding regions of the SIRT1 gene. In particular, SNPs g.-382G > A and g.-274C > G were located in the promoter region of the gene. Later, Gui et al. (2014, 2015) reported many noncoding mutations in the 3${}^{\prime }$UTR of the SIRT1 gene (g.25764G > A, g.25846A > G, g.25868T > C, g.25751A > C). Some of the abovementioned SNPs were found to be associated with various growth and carcass traits in Nanyang, Qinchuan and Luxi cattle breeds (Li et al., 2013a, b; Gui et al., 2014, 2015; Liu et al., 2017).

The transversion g.-274C > G may modulate SIRT1 promoter activity, causing the disruption of several transcription factor binding sites. In detail, in the presence of the C allele, the binding site for the CDE (cell-cycle-dependent element) was generated; this binding site was abolished when the G allele occurred (Li et al., 2013a, b). Based on the biological role of SIRT1 in metabolism regulation and mammary gland development, it is possible to suppose an association between SIRT1 polymorphisms and milk production traits. Thus, the aims of the present study were to estimate the allele and genotype frequencies at the SIRT1 g.-274C > G locus and to investigate the possible relationship among different genotypes and some milk production traits in a sample of cows belonging to the Agerolese breed. Moreover, the potential relationship between different genotypes and some reproduction traits was explored.

Agerolese cattle are a dual-purpose (milk and meat) autochthonous Italian breed reared in the Campania region. They ere established on the Lattari mountains during the 19th century starting from an autochthonous nucleus of Podolica cows crossed with Brown Swiss, Dutch Friesian and Jersey bulls. After the Second World War, the Agerolese breed underwent genetic contamination with Italian Brown and Holstein Friesian cattle (Felius, 1995). The current Agerolese cattle population is very small: to date there are 208 animals, 181 of which (147 females and 34 males) are enrolled in the birth register managed by the Italian Breeders Association (AIA, 2014). The milk produced by Agerolese cows has excellent organoleptic properties. Presently, this breed is used almost exclusively for milk production which is mainly used for butter and cheese production such as “fior di latte” (stretched curd) and “Provolone del Monaco”. a cheese recognized by the Protected Designation of Origin (reg. CE no. 121/2010) and the Slow Food Foundation for Biodiversity (Peretti et al., 2013; Selvaggi et al., 2017).

## 2 Material and methods

### 2.1 Animals

A total of 90 cows belonging to the Agerolese breed were included in this study. They were the progeny of 15 Agerolese sires with the number of half-sibs ranging from 3 to 6. Animals were maintained at a single farm located in the Campania region, in southern Italy. All the cows were fed with the same lactation diet, according to the energy recommendations for lactating cows, being able to graze for about 8–10 h after the morning milking. They had free access to water. The animals were milked twice a day. Data concerning milk production traits during all of lactation for each animal in the first, second and third lactation were obtained from monthly records provided by the National Breeders Association. In particular, data concerning the daily milk yield (DMY, kg), the fat and protein percentage (FC, PC), the peak daily milk yield (PY, defined as the highest daily milk volume produced for each animal, kg), the lactation length (LL, days), the 305-day milk yield (305MY, kg), and the 305-day fat and protein percentage (305FC, 305PC) were used in the present study. Moreover, the 305 fat/protein ratio (305FC/PC) was calculated. Thus, our dataset contained information concerning whole lactations and standard (305 days) lactations measured in 4 consecutive years (2010–2014) for each cow. Detailed information was available for some reproductive traits and was used to determine age at first calving (days) and calving intervals (days) for each animal.

### 2.2 Polymorphism determination

Individual blood samples for DNA genotyping were collected from all the cows on K3-EDTA tubes and stored at -25 ${}^{\circ }$C. Genomic DNA was isolated from whole blood using ZR Genomic DNA II KitTM (Zymo Research). After genomic DNA isolation, all the samples were genotyped for the g.-274C > G gene polymorphism using the PCR–RFLP technique. The g.-274C > G polymorphism, located in the promoter region of the bovine SIRT1 gene was determined as previously described by Li et al. (2013a). The g.-274C > G polymorphism is detectable by enzymatic digestion with SmaI (Li et al., 2013a, b).

**Table 1:** Observed and expected numbers and percentages (in brackets) of genotypes at the g.-274C < G locus, allele frequencies and population genetic indices in the sample of Agerolese cows.

					Allele							
	Genotype frequencies				frequencies		$X^{2}$	Ho	He	$N_{e}$	PIC	$F_{\mathrm{IS}}$
	CC	GC	GG		C	G	HWE					
Observed	29	36	25		0.522	0.47	3.54	0.600	0.400	1.996	0.375	0.198
	(32.22 %)	(40.00 %)	(27.78 %)				P=0.06					
Expected	24.55	44.91	20.54									
	(27.28 %)	(49.90 %)	(22.82 %)									

HWE, Hardy–Weinberg equilibrium; Ho, gene homozygosity; He, gene heterozygosity; $N_{e}$, effective allele number; PIC, polymorphism information content; $F_{\mathrm{IS}}$, fixation index.

The following primers were used to amplify a fragment of 273 bp; SIRT1F (forward): 5${}^{\prime }$ – GTA TAG TCC ACG GGG TTA CAG – 3${}^{\prime }$; SIRT1R (reverse): 5${}^{\prime }$ – CCA AAC TTG TCT TTC AGA GTC – 3${}^{\prime }$. The SIRT1 gene fragment was amplified using 34 amplification cycles of 94 ${}^{\circ }$C for 1 min, 56 ${}^{\circ }$C for 1 min and 72 ${}^{\circ }$C for 1 min. The 273 bp product was digested with SmaI restriction endonuclease (CCC↓GGG) and then analyzed on a 3 % agarose gel stained with ethidium bromide in a TBE buffer.

### 2.3 Statistical analysis

The allele frequencies were calculated by simple allele counting (Falconer and Mackay, 1996). The possible deviations of genotypic frequencies from expectation under Hardy–Weinberg equilibrium were tested by a chi-square test. Population genetic indices, such as gene heterozygosity (He), gene homozygosity (Ho), effective allele numbers ($N_{e}$) and fixation index ($F_{\mathrm{IS}}$), were performed by POPGENE32 software version 1.32 (Yeh et al., 2000). Moreover, polymorphism information content (PIC) was calculated according to Botstein et al. (1980).

A mixed model for repeated measures implemented with the SAS software (SAS 9.2 Institute, Inc., Cary, NC; SAS, 1999) was used to detect a possible relationship between the SIRT1 genotypes and performance traits under study. Data were considered as repeated measures and the correlations between the measures in the same individual were considered in the statistical model. The model included SIRT1 genotype and lactation number as fixed effects: the random effect of the sire, the random animal effect and the residual error term. The values were considered significant at P<0.05 and presented as least squares means ± standard errors.

**Table 2:** Frequencies of C and G alleles and population genetic indices in Agerolese and in different cattle breeds as observed by other authors. Allele frequencies are shown in increasing order for the C allele.

	Allelic					
Breed	frequencies		He	$N_{e}$	PIC	References
	C	G				
Chinese Red Steppe	0.447	0.553	0.494	1.978	0.372	Li et al. (2013a, b)
Agerolese	0.522	0.478	0.400	1.996	0.375	Present work
Qinchuan	0.531	0.469	0.498	1.992	0.374	Li et al. (2013a, b)
Nanyang	0.651	0.349	0.454	1.833	0.351	Li et al. (2013a, b)
Jiaxian	0.653	0.347	0.453	1.829	0.350	Li et al. (2013a, b)
Luxi	0.744	0.256	0.381	1.615	0.308	Li et al. (2013a, b)
Luxi	0.769	0.231	0.355	1.550	0.292	Liu et al. (2017)

**Table 3:** Means and standard error of productive and reproductive traits in Agerolese cows with different genotypes at g.-274C > G.

Traits	Genotypes		
	CC	CG	GG
DMY (kg)	$15.04^{b}\pm 0.69$	16.58±0.61	$17.19^{a}\pm 0.65$
FC (%)	$3.84^{a}\pm 0.05$	3.62±0.03	$3.55^{b}\pm 0.06$
PC (%)	3.31±0.03	3.28±0.02	.28±0.03
LL (days)	$337.33^{A}\pm 9.90$	$304.60^{A}\pm 8.31$	$259.87^{B}\pm 11.15$
PY (kg)	$22.49^{b}\pm 0.65$	$25.29^{a}\pm 0.56$	$25.79^{a}\pm 0.64$
305MY	$4165.67^{b}\pm 198.15$	$5018.30^{a}\pm 166.18$	4378.83±187.27
305FC (%)	3.81±0.05	3.60±0.03	3.55±0.06
305PC (%)	3.26±0.03	3.26±0.02	3.27±0.03
305FC/PC	1.17±0.01	1.11±0.01	1.09±0.01
Age at first calving (days)	$848.23^{a}\pm 28.65$	786.82±21.79	$772.50^{b}\pm 26.42$
Calving period (days)	$454.85^{\mathrm{Aa}}\pm 15.18$	$401.30^{b}\pm 12.42$	$398.97^{B}\pm 13.99$

${}^{A,B}$ = P<0.01; ${}^{a,b}$ = P<0.05; DMY, daily milk yield; FC, fat content; PC, protein content; LL, lactation length, PY, peak daily milk yield; 305MY, 305-day milk yield lactation; 305FC, 305-day fat content; 305PC, 305-day protein content; 305FC/PC, 305-day fat-to-protein-content ratio.

## 3 Results and discussion

### 3.1 Gene frequency

The nuclease cuts the 273 bp amplification product into 235 and 38 bp fragments for allele G, while allele C remains uncut. The following DNA restriction fragments were expected: 235 and 38 bp for the GG genotype; 273, 235 and 38 bp for the CG genotype; and 273 bp for the CC genotype. As shown in Table 1, the investigated population was found to be polymorphic at the g.-274C > G locus: in particular, 29 individuals out of 90 were genotyped as CC, 25 as GG and 36 were heterozygotes. Thus, the frequencies of C and G alleles were 0.522 and 0.478, respectively. The expected genotype frequencies, calculated according to the Hardy–Weinberg equilibrium, were 27.28 % (CC), 49.90 % (GC), and 22.82 % (GG). The calculated chi-square value was 3.54 (degree of freedom = 1), indicating a Hardy–Weinberg equilibrium in the population (P=0.06). Table 1 also illustrates the calculated values of the genetic indices. $F_{\mathrm{IS}}$ is a measure of the deviation of genotypic frequencies from panmictic frequencies in terms of heterozygous deficiency or excess. Negative $F_{\mathrm{IS}}$ values indicate heterozygote excess and positive values indicate heterozygote deficiency when compared with Hardy–Weinberg equilibrium expectations. In the Agerolese population a slight excess of homozygosity at the considered locus was found ($F_{\mathrm{IS}}=0.198$). PIC is a parameter indicative of the degree of informativeness of a marker. The PIC value may range from 0 to 1. In the studied population, the PIC value was 0.375. According to the classification of PIC (low polymorphism if PIC value < 0.25, median if 0.25 < PIC value < 0.50 and high if PIC value > 0.50), the examined population possesses an intermediate polymorphism level at the considered locus. Moreover, the calculated $N_{e}$ indicates a good level of genetic variability in the Agerolese cattle breed at the studied locus. Table 2 summarizes the previous findings on allele frequencies and genetic indices at the SIRT1 c.-274C > G locus in other breeds. This SNP was found to be polymorphic in all the investigated breeds. The C allele is the most frequent in all these populations, except for Chinese Red Steppe. Moreover, all the breeds possess a high effective allele number (ranging from 1.550 to 1.996) and a moderate value of PIC (ranging from 0.292 to 0.375).

### 3.2 Effects of polymorphism on productive and reproductive traits

Data reported in Table 3 show the effects of the g.-274C > G polymorphism on milk production traits and some reproductive traits. Concerning milk production performances, significant differences between genotypes were found in DMY, FC, LL, PY and 305MY. In particular, when compared with the other genotypes, GG individuals produced a greater quantity of milk in terms of DMY (17.19 kg day${}^{-1}$ vs. 15.04 kg day${}^{-1}$ for GG and CC, respectively; P<0.05); however the difference between GG and CG was found not to be significant in statistical analysis. On the other hand, CC and CG animals showed a longer lactation when compared to GG genotypes (337.33 and 304.60 days vs. 259.87 days, respectively; P<0.01). Moreover, PY of cows carrying the CC genotype was lower when compared with the CG and GG ones (22.49 kg vs. 25.29 and 25.79 kg, respectively; P<0.05). Milk quality was significantly affected only in terms of FC (3.84 % vs. 3.55 % for CC and GG cows, respectively; P<0.05), with no difference in protein content. When production data during standard lactation are considered, no differences were found concerning the quality of milk. Moreover, CG animals yielded a greater amount of milk when compared to cows belonging to CC genotype (5018.30 kg vs. 4165.67 kg; P<0.05). Nevertheless, this last result needs more attention due to the short lactation length of GG animals (less than 305 days). Although GG animals produced less milk during the standard lactation when compared to CG animals, this difference was not significant in statistical analysis.

The effect of the g.-274C > G genotype on age at first calving and calving period was significant. In detail, GG cows had a younger age at first calving than CC ones (772.50 days vs. 848.23 days; P<0.05). Animals having a CG genotype showed an intermediate age at first calving, although the differences to the other genotypes were not significant in statistical analysis. Finally, cows carrying the GG genotype had a shorter calving interval when compared with CC individuals (-55.88 days; P<0.01), and also the difference between CC and CG genotype was found to be significant (454.85 days vs. 401.30 days, respectively; P<0.05). No significant result was obtained by comparing the length of the calving period of CG and GG animals.

To the best of our knowledge, no other studies on the potential effect of SIRT1 or SmaI polymorphism on milk performances and reproductive traits are available in the literature. On the basis of our results, GG animals were more productive in terms of DMY although they showed a shorter lactation period that is the reason for the lower 305MY. Nevertheless, this aspect can be balanced out considering the more advantageous reproductive traits of GG cows.

To date, only few studies have been conducted in order to find a possible relationship between SIRT1 gene polymorphisms and livestock performance traits. Nevertheless, the SIRT1 gene seems to be a potentially useful genetic marker for carcass and body measurement characteristics (Li et al., 2013a, b; Gui et al., 2014; Liu et al., 2017). In particular, Li et al. (2013a, b) found a positive association between the c.-274C > G polymorphism and growth traits in Nanyang cattle suggesting that the presence of the G allelic variant could be responsible for a decreased gene promoter activity that, in turn, is responsible for the improved growth traits observed in CG and GG animals. Considering this last aspect, it is possible to suppose that CG and GG animals may reach the minimum liveweight at mating early. Thus, the lower age at first calving observed in CG and GG individuals in the present study may be explained by an improved somatic precocity.

## 4 Conclusion

Undoubtedly, sirtuins are a very interesting family of protein, playing a key role in endocrine signaling. Considering the specific involvement of SIRT1 in the glucose and fat metabolism, to study the variability of SIRT1 gene may offer the possibility to contribute to improving livestock production. This is the first report on the possible association between different genotypes at the SIRT1 g.-274C > G locus and milk productive and reproductive traits in bovine species. Although other studies should be carried out on sirtuins and their genetic variability, our results are promising and should encourage scientists to further investigate the effect of genetic polymorphism of sirtuins on milk performance and reproductive traits.

## Data Availability

The data sets are available upon request from the corresponding author.
